# Complete remission of platinum-refractory primary Fallopian tube carcinoma with third-line gemcitabine plus cisplatin: A case report and review of the literature

**DOI:** 10.3892/ol.2013.1232

**Published:** 2013-03-06

**Authors:** QIUYI XU, NONG XU, WEIJIA FANG, PENG ZHAO, CHENYU MAO, YULONG ZHENG, HAIBO MOU

**Affiliations:** Department of Medical Oncology, The First Affiliated Hospital, School of Medicine, Zhejiang University, Hangzhou, Zhejiang 310003 P.R. China

**Keywords:** primary Fallopian tube carcinoma, gemcitabine, refractory, complete remission

## Abstract

Primary Fallopian tube carcinoma (PFTC) is a rare but highly aggressive disease. Currently, treatments are similar to those used in epithelial ovarian carcinoma (EOC), however, there are distinct differences between the two diseases. PFTC tends to recur in the retroperitoneal nodes and distant sites more often than EOC. Limited literature with regard to effective agents in platinum-resistant and -refractory (Pt-R) disease exists, particularly after two lines of consecutive treatment. In this case report, a 47-year-old female with PFTC exhibited recurrence in the liver after postoperative chemotherapy. The patient received paclitaxel and cisplatin combination as first-line chemotherapy and topotecan as a second-line treatment, which is considered platinum-refractory. After the second-line treatment failed, this patient received a gemcitabine plus cisplatin combination as third-line chemotherapy for a total of 6 cycles. The liver metastases regressed rapidly and completely. The patient’s progression-free survival (PFS) was 10 months and overall survival (OS) was 45 months. In conclusion, gemcitabine and cisplatin combination is an effective regimen for refractory PFTC even after the failure of two previous lines of consecutive chemotherapy and this warrants further independent investigation.

## Introduction

Primary Fallopian tube carcinoma (PFTC) is a rare but highly aggressive disease that accounts for 0.14–1.8% of gynecological malignancies (1). Due to a lack of specific symptoms and signs, PFTC is rarely identified preoperatively and is usually first diagnosed at the time of surgery or by a pathologist. Surgical resection is the primary treatment for PFTC and the best chance of cure, and is usually followed by postoperative chemotherapy. The majority of patients experience recurrence or metastasis involving the pelvis, upper abdomen, retroperitoneal nodes and distant sites and receive platinum-taxane combinations as first-line chemotherapy. Second-line treatment is normally based on the same guidelines used for epithelial ovarian carcinoma (EOC), as the two diseases have similar histological and biological features. Owing to the aggressiveness of the malignancy, there are limited data on the exact activity of regimens for third-line treatment in patients with advanced PFTC.

Gemcitabine is a pyrimidine antimetabolite that demonstrates modest activity in platinum-refractory or -resistant (Pt-R) EOC as second-line chemotherapy. Phase II studies have identified response rates (RRs) of 20% with single-agent gemcitabine (2) and several others have shown a 15.8–70% RR with platinum and gemcitabine combination (3).

In this study, we report a complete remission (CR) of refractory PFTC with gemcitabine and cisplatin combination after the failure of two previous lines of consecutive chemotherapy; an outcome which has not been reported previously.

This study was approved by the Institutional Ethics Committee of The First Affiliated Hospital, School of Medicine, Zhejiang University, Hangzhou, China. Written informed consent was obtained from the patient.

## Case report

### Clinical presentation and diagnosis

A 47-year-old female presented with complaints of increasing abdominal pain on December 4, 2008. The patient underwent an ultrasound, which revealed ovarian cysts, and the initial serum cancer antigen (CA)-125 level was 412 U/ml (normal level, <35 U/ml). An exploratory laparotomy was performed, including a total hysterectomy, bilateral salpingo-oophorectomy, partial omentectomy and pelvic lymph node dissection. Final staging and pathology were consistent with poorly differentiated serous papillary carcinoma of the right Fallopian tube, at International Federation of Gynecology and Obstetrics (FIGO) stage IIIC. Immunohistochemical staining of the tumor was positive for cytokeratin 7, CA-125, cytokeratin 5/6 and Mes and negative for cytokeratin 20, CEA, CgA, Syn and calretinin.

### Treatment and clinical course

The patient received postoperative chemotherapy consisting of paclitaxel (175 mg/m^2^) and carboplatin (AUC, 5), administered at 3-week intervals for a total of 6 cycles. CA-125 levels decreased to 13 U/ml. No objective evidence of residue disease existed. At a scheduled evaluation 14 months later, CA-125 levels were elevated to 214 U/ml and computed tomography (CT) scans revealed multiple liver metastases. The patient received 2 cycles of paclitaxel and carboplatin combination as first-line chemotherapy, however, CA-125 levels elevated rapidly. Subsequently, according to the Response Evaluation Criteria in Solid Tumors (RECIST), disease progression was evident on radiological assessment (April 20 2010). Considering this was a platinum-refractory disease, the patient underwent 2 cycles of topotecan as a second-line treatment. However, CA-125 remained at an elevated level and CT scans showed the liver metastatic tumor to be enlarged. After the two lines of consecutive chemotherapy, a combination of 1000 mg/m^2^ gemcitabine on days 1 and 8 and 75 mg/m^2^ cisplatin on day 1 was administered as third-line chemotherapy, at 3-week intervals. After 3 cycles, the CA-125 level was 21 U/ml and CT scans showed CR of the liver metastases. A total of 6 cycles of chemotherapy was completed. Liver metastases were in CR for a further 6 months. Progression-free survival (PFS) reached 10 months and the overall survival (OS) was 45 months.

## Discussion

PFTC is a rare gynecological neoplasm and its incidence rate is 4.1/1 million females each year. Among females aged 65–69 years, incidence rates increased significantly by 3.8% per year between 1998 and 2003 (4). The most common symptoms of PFTC are postmenopausal vaginal bleeding and discharge (35–60% of patients), followed by abdominal pain (30*–*49%) and abdominal masses (12–61%). The main histological subtype of PFTC is serous adenocarcinoma (50*–*80%). Staging is based on the surgical findings at laparotomy. In general, 20*–*25% of patients have stage I, 20% have stage II, 45*–*50% have stage III and 5*–*10% have stage IV disease (5). CA-125 is a useful tumor marker for PFTC. It is an early and sensitive marker for tumor progression during post-treatment follow-up, where it has been reported that the lead time (elevated CA-125 levels prior to clinical or radiological diagnosis of recurrence) is 3 months (range, 0.5–7 months) (6).

The optimal therapeutic strategy for PFTC has not yet been established due to its rarity, thus treatments follow those used in EOC, owing to the similarities between the two diseases. However, there are several distinct differences between PFTC and EOC. PFTC tends to be more frequently diagnosed at an early stage, due to abdominal pain secondary to tubal distension, and tends to recur more often in the retroperitoneal nodes and distant sites (7). The 5-year OS rate of PFTC is 59% for stages I and II and 19% for stages III and IV (8). Due to the increased incidence rates, different characteristics and poor diagnosis of PFTC compared with EOC, investigations focusing on the optimal therapeutic strategy and effective agents for PFTC are required.

Current studies suggest that surgery is the primary treatment for patients with PFTC. Optimal debulking with removal of the maximum amount of tumor possible is warranted in patients. After surgical resection, postoperative chemotherapy is usually suggested due to the high risk of distant metastases and postoperative radiotherapy is no longer recommended (9). Platinum-taxane combinations are considered the standard treatment (10), where the median time to disease progression was 84 months for patients with early stage disease. For advanced disease, study data from small series show that platinum-based regimens achieve an objective RR (ORR) in 53–92% of patients (11). Platinum- and paclitaxel-based regimens in first-line chemotherapy have a high RR in 87.5–93% of patients (10,12,13). Second-line treatment was administered according to EOC, based on platinum-free intervals. Platinum-sensitive patients (relapse after 6 months) are usually retreated with platinum with or without paclitaxel, whereas platinum-refractory (progression during platinum-based therapy) or platinum-resistant (relapse within 6 months) patients are treated with non-platinum agents. In Pt-R disease, the prognosis is always poor.

Literature on the active treatment for Pt-R PFTC is limited. Tresukosol *et al*(14) reported a patient who achieved CR with high-dose paclitaxel after pretreatment with platinum. Dunton and Neufeld (15) used topotecan to treat a patient who previously received carboplatin and paclitaxel and demonstrated clinical CR. With regard to EOC, several agents have emerged which show definite activity in recurrent EOC, including topotecan, pegylated liposomal doxorubicin, prolonged oral etoposide and gemcitabine. Matsuo *et al*(16) analyzed previous clinical trials and identified that these regimens have similar RRs, thus no standardized treatment exists for Pt-R EOC.

Several studies showed that gemcitabine had modest activity as a second-line treatment for Pt-R EOC, with an ORR that varies from 11–29%. Furthermore, the combination of gemcitabine with cisplatin has synergistic activity with regard to platinum-DNA adduct formation in preclinical studies (17). Clinical data show that the ORR varies from 16–64% and PFS varies from 4.9–6.7 months (18–22). Rose (23) showed that gemcitabine is able to reverse platinum resistance in platinum-resistant ovarian and peritoneal carcinoma. In this case, the patient had platinum-refractory PFTC and the second-line treatment of topotecan failed. Griffiths *et al*(24) demonstrated that failure to achieve disease control after two lines of chemotherapy suggests that further anticancer therapy is unlikely to be effective and prognosis is poor in EOC. However, in this case, we observed a CR which lasted for 10 months in third-line chemotherapy with gemcitabine and cisplatin combination and OS was prolonged significantly.

This is the first case of clinical CR with gemcitabine and cisplatin combination after failing in two previous lines of consecutive chemotherapy in recurrent PFTC. Few agents have shown activity in recurrent disease, particularly following multiple lines of treatment. This case suggests that gemcitabine plus cisplatin is an effective regimen for Pt-R PFTC to prolong survival time, even after multiple lines of treatment. Furthermore, the therapeutic strategy for PFTC may require a different approach to EOC, as aggressive treatment is important for PFTC patients and its increasing incidence means that further independent study on the optimal treatment is warranted.

## Figures and Tables

**Figure 1:**
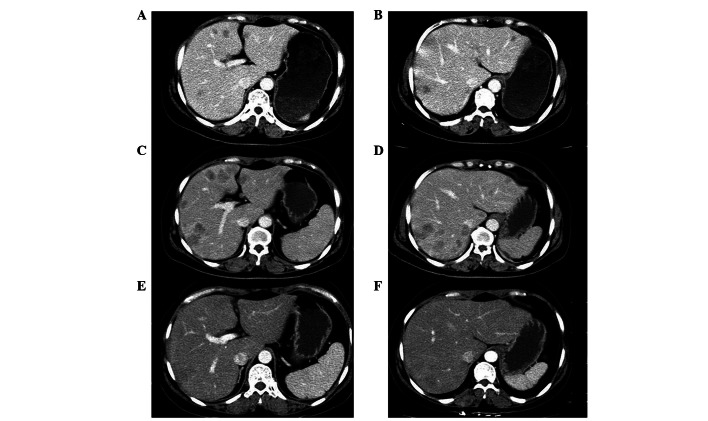
CT scans of liver metastases in a patient with a poorly differentiated serous papillary carcinoma of the right Fallopian tube. (A and B) At diagnosis of the metastases (March 2010); (C and D) after 2 cycles of second-line chemotherapy with topotecan (August 2010); (E and F) after 3 cycles of third-line treatment with gemcitabine plus cisplatin (October 2010). CT, computed tomography.
